# Distinct sensory atypicalities bridge the gap between brain chemistry and motor dysfunction in autism

**DOI:** 10.1038/s41398-026-04036-z

**Published:** 2026-05-08

**Authors:** Mingrun Shi, Jason L. He, Helen Powell, Oliver Lack, Georg Oeltzschner, Alyssa Deronda, Deana Crocetti, Ericka L. Wodka, Richard A. Edden, Jonathan O’Muircheartaigh, Stewart H. Mostofsky, Nicolaas A. J. Puts

**Affiliations:** 1 Department of Forensic and Neurodevelopmental Sciences, Institute of Psychiatry, Psychology, and Neuroscience, King’s College London, London, UK; 2 Russell H. Morgan Department of Radiology and Radiological Science, The Johns Hopkins University School of Medicine, Baltimore, MD US; 3 F. M. Kirby Research Center for Functional Brain Imaging, Kennedy Krieger Institute, Baltimore, MD US; 4 Center for Neurodevelopmental and Imaging Research, Kennedy Krieger Institute, Baltimore, MD US; 5 Center for Autism Science, Services and Innovation, Kennedy Krieger Institute, Baltimore, MD US; 6 MRC Centre for Neurodevelopmental Disorders, King’s College London, London, UK; 7 Department of Neurology, The Johns Hopkins University School of Medicine, Baltimore, MD US; 8 Department of Psychiatry and Behavioral Sciences, The Johns Hopkins University School of Medicine, Baltimore, MD US

**Keywords:** Human behaviour, Molecular neuroscience

## Abstract

Sensory and motor difficulties are common in autism. Altered excitation-inhibition (E-I) balance is a putative framework for understanding atypical sensory and motor function. We investigated whether sensory differences of autism mediate motor difficulties of autism via differences in E-I balance. 106 children were included in the study (Autism n = 44, Typical development children (TDC) n = 62, age 10.32 ± 1.49). E-I balance was assessed through magnetic resonance spectroscopy (MRS), quantifying Glutamate and Glutamine (Glx) and Gamma-Aminobutyric Acid (GABA) in primary sensorimotor cortex (SM1) and thalamus (Thal). Sensory function was evaluated using both objective vibrotactile perceptual sensitivity assessments and subjective parent ratings via the Sensory Experience Questionnaire (SEQ). Motor ability was assessed objectively through the Movement Assessment Battery for Children-second edition (MABC-2) and the Physical and Neurological Examination for Subtle Signs (PANESS). Our findings reveal that lower sensory reactivity and lower tactile thresholds are both predictive of better motor ability (R_sig_ range between 0.32 and 0.57) with higher sensory scores reflecting poorer sensory filtering predicting worse motor function (R_sig_ range −0.22 and −0.63). We identified significant associations between MRS-measured Glx and GABA+ levels and sensory reactivity (p < 0.001). Importantly, sensory reactivity sub-scores were found to fully mediate E-I balance to motor associations in domain-specific patterns: Hyper-reactivity mediated the impact of SM1 Glx levels, while hypo-reactivity mediated the impact of SM1 GABA levels. Additionally, sensory seeking mediated the impact of Thalamic GABA levels with all indirect paths ab p < 0.01. These results propose a model where regional metabolite-specific markers of E-I balance explain patterns of autism-associated sensory and motor difficulties, and where subsequently, distinct sensory phenotypes differentially mediate metabolite-motor associations (see Graphical Abstract for detail).

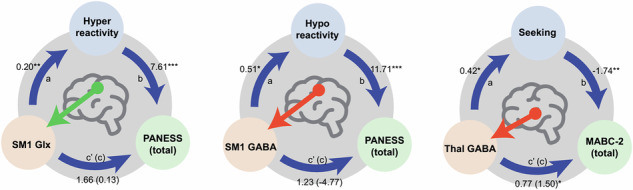

## Introduction

In addition to difficulties with social communication and restricted, repetitive behaviours and interests [1], autistic people also commonly present with both sensory [2] and motor differences [3, 4]. While there are many studies investigating the aetiology of both sensory and motor differences in autism, few studies have investigated how sensory and motor differences are correlated in autism [5–8]. This is somewhat surprising since sensory and motor development strongly overlaps, particularly in early life [9]. In the current study, we concurrently assessed sensory function and motor ability in autistic and non-autistic children. In an iterative manner, we first investigated the degree to which sensory function and motor ability were related, and whether these associations differed between autistic and neurotypical (TDC, Typically Developing Children) individuals. Participants also underwent magnetic resonance spectroscopy (MRS) in which we obtained indirect markers of excitation-inhibition (E-I) balance from sensory- and motor-related regions of the brain, in the form of brain Gamma-Aminobutyric Acid (GABA) and Glutamate and Glutamine (Glx). We investigated whether, as with the sensory differences of autism [10], the motor difficulties of autism can also be explained by differences in markers of E-I balance.

## The co-development of sensory and motor functions

Sensory and motor systems are inherently linked. The integration of bottom-up sensory information with top-down planned motor actions is necessary for fluid and accurate motor control [9]. Motor actions themselves can also influence how we process sensory information from the external environment. Given the link between sensory and motor systems, one might hypothesise that alterations to one system will result in alterations of the other, particularly if these alterations are present in early development. Indeed, the sensory differences of autism are apparent in early life [11], and can precede the presentation of other autistic traits, including difficulties with motor control.

## Sensory differences and their link to motor difficulties in autism

In the current study, we differentiate sensory differences between tactile perceptual differences and differences in sensory reactivity (as defined in the DSM-5 and Sensory Experiences Questionnaire (SEQ-3)). Perceptual sensitivity describes how well individuals can detect and discriminate between the low-level characteristics of sensory information (i.e., primary sensory properties such as stimulus intensity, processed at lower hierarchical levels of the sensory processing system, in contrast to higher-order processes involving cognition or emotion), such as the detection of a single tactile stimulus or the differentiation between two near-identical tactile stimuli. Differences of perceptual sensitivity in autism, typically quantified using objective assessments, have long been reported, with differences being identified in audition [12], vision [13], touch [14], taste [15] and olfaction [16]. Sensory reactivity patterns represent individuals’ emotional and reactive responses to sensory stimulations, including hyper-, and hypo-reactivity as well as sensory seeking and repetitive behaviours, which are typically assessed using subjective questionnaires and rating scales. Our previous studies focusing on sensory alterations in various neurodevelopmental conditions found difficulties in tactile discrimination in the autism group being exclusively correlated with autism symptoms, a significant positive correlation between static detection threshold with both SEQ Hyper-responsiveness and Hypo-responsiveness was also reported [17].

Despite there being an obvious link between sensory and motor systems in development, very few studies have investigated their association in autism [5–8]. Existing studies which have tested the relationship between sensory and motor differences in autism have used self- or parent reports of affective reactivity to sensory input [5–8] rather than quantitative measures of perceptual sensitivity. Moreover, questionnaires assessing sensory reactivity, typically cover all sensory domains and do not account for how different sensory domains might differentially contribute to motor control. In addition, motor control might be expected to be more tightly linked with sensory perception than with higher-order emotional responsivities. However, as it stands, the relationship between perceptual sensitivity and motor ability in autism remain unknown.

One potential framework to examine the association between sensory and motor differences is that of altered excitation-inhibition balance in autism [18]. This theory proposes that the biology of autism is driven by an imbalance between excitatory and inhibitory neurotransmission in the brain. Evidence from mouse models and human studies emphasise the impact of E-I balance on autistic traits. Differences have been found to support differences in excitation and inhibition through genetic studies showing differences in GABAergic and glutamatergic signalling [19], molecular and animal studies showing that synaptic and developmental differences in neuron function contribute to sensory and social differences [20–22] and human MRS and EEG studies showing differences in proxy markers of E-I [23–25]. The direction of the differences varies across brain regions, but generally an increase in excitation and decrease in inhibition are found in autism. In prior work we and others have shown that differences in both tactile perceptual sensitivity and sensory reactivity could be due to altered E-I balance in autistic children [10] as measured using MRS. Specifically, higher Glx levels have been associated with increased sensory hyper- and hyporeactivity. Additionally, altered markers of E-I balance have also been associated with motor difficulties in autistic individuals, with higher GABA levels in primary motor cortex associated with poorer performance on muscle strength related movement and lower GABA levels in supplementary motor area associated with poorer performance with motor coordination tasks [26]. MRS is a non-invasive technique allowing for the measurement of in vivo metabolite levels. In the current study we use edited MRS to measure GABA and glutamate levels (at 3 T field strength, the GABA and glutamate signals are difficult to reliably separate from overlapping macromolecular and glutamine signals, respectively, and therefore the composite measures of GABA+macromolecules and glutamate+glutamine levels are typically referred to as GABA+ and Glx [glutamate + glutamine] [27]).

## The current study

The aim of this study was to investigate the link between sensory differences, motor difficulties and E-I balance in autistic (n = 44) and TDC (n = 62). GABA+ and Glx levels were used as the proxy markers of E-I balance. We propose that GABA+ and Glx levels in the SM1 and Thal will not be directly related to motor ability in autism; this contrasts with prior findings revealing direct correlations with sensory differences [10]. Instead, we predict that the associations between GABA+ and Glx and motor ability will be mediated through sensory differences. That is, we hypothesize the following indirect pathways: brain metabolite levels (X; GABA+ and Glx in SM1 and Thal) → (M) sensory functioning → (Y) motor ability (Supplementary Fig. 1). We hypothesized that: 1) MRS markers of E-I balance would significantly correlate with measures of sensory function, both objective measures of tactile perceptual sensitivity and subjective measures of sensory reactivity (consistent with [10]), 2) Measures of sensory function would correlate with measures of motor function, and 3) MRS markers of E-I balance would not directly correlate with objective measures of motor ability; rather, a significant association would be mediated by sensory function.

## Participants and methods

The data presented in this study fall under the ethical approval of the Kennedy Krieger Institute and the Johns Hopkins School of Medicine Institutional Review Boards. A caregiver of each child who participated in testing provided written informed consent on the child’s behalf and all children provided oral assent. All methods were performed in accordance with the relevant guidelines and regulations.

### Participants

Children (N = 106) were included in the study if they had completed both the Movement Assessment Battery for Children-second edition (MABC-2) and the Physical And Neurological Examination for Subtle Signs (PANESS), and at least one measure of tactile perceptual sensitivity. Of the 106 children, 44 were autistic and 62 were TDC, with ages ranging from 8–14 years, and Wechsler Intelligence Scale for Children Fifth edition (WISC5) Full Scale Intelligence Quotient (IQ) scores ranging from 70–146. All children included in this study underwent MRS to determine metabolite levels relevant to E-I balance.

Autistic participants met diagnostic criteria for an autism spectrum disorder (ASD) on either the fourth (DSM-IV; [28]) or fifth edition (DSM-5; [29]) of the Diagnostic and Statistical Manual of Mental Disorders. Diagnoses were confirmed using the Autism Diagnostic Observation Schedule - Second Edition (ADOS-2; [30]). The WISC-5 [31] was used to determine cognitive and intellectual ability. Handedness was determined using the Edinburgh Handedness Inventory [32]. Children who had an identifiable genetic cause of autism, such as Fragile X syndrome, and other neurological disorders were excluded. Children with full-scale IQ scores below 80 were excluded to ensure adequate task comprehension, except in cases where participants demonstrated a 12-point or greater discrepancy. For these exceptional cases, participants were retained if either their Verbal Comprehension Index or Perceptual Reasoning Index was ≥ 80, with the lower of the two indices being ≥ 65. Autistic children on stimulant medication were instructed to discontinue stimulant medication on and before the day of participation (n = 14) but children were allowed to take other psychotropic medications requiring extended washout. (Autism: alpha-2 adrenergic agonists n = 13, selective serotonin reuptake inhibitor (SSRI) n = 7, dopamine receptor blockers n = 2, sleep medication n = 2, serotonin–norepinephrine reuptake inhibitor/norepinephrine reuptake inhibitor (SNRI/NRI) n = 1, atypical antidepressants n = 1, hormone medication n = 1, type of medications were not collected n = 6; TDC: allergy medication n = 13, non-brain medication n = 1, sleep medication n = 1, type of medication were not collected n = 4.) The Diagnostic Interview for Children and Adolescents – Fourth Edition (DICA-IV; [33]) was used to ensure that children in the TDC group did not meet criteria for any neurodevelopmental conditions.

### Measures

#### Sensory

##### Tactile perceptual sensitivity

Tactile perceptual sensitivity was assessed using a battery of vibrotactile psychophysical tasks. The full details of this battery have been detailed in our earlier work [17, 34]. From these tasks we additionally calculated various indices to reflect differences in performance between associated tasks. For all thresholds, lower values suggest better sensitivity. For all difference indices, higher values suggest more inhibition during sensory processing. Completion rates and a visual schematic for each task can be found in Supplementary Fig. 2 and Supplementary Fig. 3.

##### Sensory experience questionnaire

Sensory reactivity was assessed using the Sensory Experience Questionnaire (SEQ; [35]). SEQ is a caregiver-report measure designed to characterize sensory features and assess patterns of hyper- and hypo-reactivity as well as sensory seeking behaviours in autistic children. The reliability and validity of this instrument have been established specifically for use with autistic populations [36]. Mean subscale scores were used for reporting SEQ subscale. Lower SEQ scores suggest lower reactivity.

#### Motor

##### Assessment of motor abilities

MABC-2 [37] is a tool developed to assess children’s fine and gross motor skills. The assessment contains eight tasks to measure three separate areas of motor ability: Balance, Manual Dexterity, and Aiming and Catching. Raw scores in each task can be combined to produce standard scores and percentile rankings. Given that interindividual variability is reduced when converting from standard scores to percentile rankings, we used standard scores for our analyses. Higher scores on the MABC-2 reflect less motor impairment.

PANESS [38] is a norm-referenced examination for detecting subtle signs of motor impairment. The assessment is divided into three sections: Lateral Preference, Gaits and Stations, and Timed Motor. Variable scores are derived from behaviours observed during the tasks, including foot-to-hand overflow, dysrhythmia, and standard deviation from the mean. The PANESS yields four subscales: Total Timed, Total Overflow, Gaits-Stations, and Total PANESS. Higher scores on the PANESS reflect greater motor impairment. The Overflow subscale quantifies excess movement (e.g. from both contra- and ipsilateral hands), particularly extraneous movements resulting from insufficient motor control, and is scored based on overflow behaviours observed during the Gaits and Timed sections [39].

### MRS of GABA+ and Glx

#### Acquisition

**Fig. 1:**
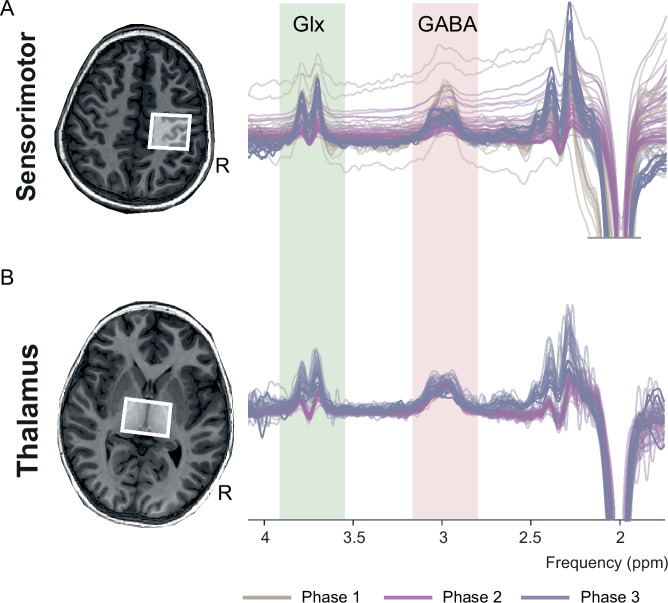
Voxel placement and spectra. **A** Voxel placement over the right primary sensorimotor cortex (SM1) in a randomly selected participant. All spectra are also presented on the right. Note that the right SM1 was selected as the vibrotactile battery was delivered to the participant’s left hand. **B** Voxel placement over the bilateral thalamus (Thal) in a randomly selected participant. All spectra are also presented on the right. Spectra colours represent the different acquisition phases (brown = phase 1, purple = phase 2 and blue = phase 3). A more comprehensive description of each of the phases is described in Supplementary Materials.

All MRS data were acquired on Philips 3 T magnetic resonance imaging scanner (Philips Healthcare, Best, The Netherlands). Data were acquired from the right SM1 and the bilateral Thal as we are performing the tactile task on left hand, see Fig. 1. We used a standard approach for data acquisition and analysis which is described in prior work [10]. Further details can be found in the Supplementary Methods. In brief, MRS was acquired in three ‘phases’ as outlined in Jason et al. (2021) [10]. This was done due to scanner upgrades and using the approach that, at the time, was deemed more appropriate. This includes macromolecule-suppressed GABA-edited MEGA-PRESS (phase 1, before upgrade, phase 2, after upgrade) and HERMES (phase 3) for simultaneous editing of GABA+macromolecules and GSH. To ensure data consistency across acquisition phases, we maintained standardized voxel placement protocols, applied identical preprocessing pipelines, and used uniform quality control criteria for all participants. MRS data were then processed using Gannet 3.1, with MRS data being pre-processed, fitted to model different metabolite and quantified for metabolite estimates, and based on the experts’ consensus recommendations [40], we report Glx (Glutamate and Glutamine) and GABA+ (GABA+macromolecules) from the MRS signal. The MRS analysis software (Gannet) output plots for each dataset were visually inspected by a member of the study team (GO, ~ 9 years of experience using edited MRS). Data themselves or the fits considered unusable. subtraction artefacts and lipid contamination in cortical voxels, were excluded from further analyses. For more detail about objective metrics for MRS data quality, please find in our earlier study [10].

## Statistical analyses

Analyses were conducted using the R programming language in R studio (version 4.0.3; [41]). Visualisation of data was conducted using the ‘*ggplot2*’ package [42]. Partial eta-squared was used to measure effect size and was estimated using the ‘*effectsize*’ package [43]. Average causal mediation effects (ACME) were used to measure the significance of mediation effect using the ‘*mediation*’ package.

This work is based on our prior work in this sample, which showed effect sizes between 0.3 and 0.5 associating sensory perception and reactivity [17], and approximately 0.3 [10] for relationships between MRS measures and sensory reactivity. Therefore, we believe the sample is sufficient for estimating significant mediation effects.

We first conducted correlation analysis to validate our hypothetical model by assessing associations between these factors (Supplementary Fig. 1). We then determined effects between the variables in the hypothetical model by conducting a series of independent linear regression analyses. Using sample sizes (n_1_, n_2_) and correlation coefficients (r_1_, r_2_) from both groups (autism and TDC), we conducted Fisher Z-transformations to determine whether the relationship between motor ability and sensory function differed between autism and TDC groups. FDR correction was applied to account for multiple comparisons.

To determine whether sensory function mediated the relationship between brain metabolite levels and motor ability, we conducted a series of simple linear regression analyses. In these linear regressions, brain metabolite levels were set as independent variables, motor abilities as dependent variables, and sensory functioning scores as mediators. We also considered the opposite direction in this mediation effect, using motor ability as mediator and sensory differences as outcome. However, these mediations were not significant (see Supplementary Materials for details). We then used average causal mediation effects (ACME) analysis within causal mediation modelling because it addresses key limitations of conventional mediation approaches, which require a significant direct relationship between brain metabolites and motor ability. ACME analysis can detect mediation effects even when this total effect is non-significant, an advantage given our findings. Additionally, ACME provides sensitivity analysis tools that allow us to assess the robustness of any mediation effects and validate the mediator’s role in our hypothesized pathway from brain chemistry through sensory processing to motor outcomes [44].

The level of the unmeasured effect in our hypothesized mediation model was tested by conducting a sensitivity analysis using bootstrapping [45]. During analyses, estimated ACME were calculated as a function of ‘rho’, the correlations between the residuals in mediators and dependent variables, which represented the level of inaccuracy of our estimation. Rho was incremented by 0.05 each time during estimation. There was no inaccuracy in estimation when rho was equal to zero, while rho at which ACME was equal to zero represented the level of inaccuracy of our estimation when there was no indirect effect in our mediation model, which was caused by other unmeasured factors. Therefore, rho when ACME was equaled to zero was used to indicate the robustness of the hypothetical mediation model, a smaller absolute value of rho indicated a higher degree of robustness of the mediation model. Mediation analyses were conducted both for the combined sample and separately within each group. For the autism group, we tested mediation pathways where sensory function scores showed significant correlations with both metabolite levels and motor abilities, meeting the prerequisite conditions for causal mediation analysis. For the neurotypical group, no mediation analyses were conducted as no sensory function scores were significantly correlated with both metabolite levels and motor abilities, likely due to bottom effects, precluding meaningful mediation testing.

## Results

Descriptive statistics and results from group comparisons are presented in Table 1. Group differences on the MABC-2 and PANESS total scores are additionally visualised in Supplementary Fig. 4. The difference in male to female ratios and age between the groups was statistically significant (p < 0.001). For this reason, analyses involving a comparison of autism and TDC groups outside of the descriptive statistics presented in Table 1 included sex and age as covariates. Group differences could be identified for full-scale IQ and the index scales of the WISC-5. We included only children with full-scale IQ scores above 80 (or Verbal Comprehension Index/Perceptual Reasoning Index ≥ 80 when significant discrepancies existed between indices), ensuring adequate task comprehension and performance while excluding individuals with intellectual disability. Additionally, we did not control for IQ as a covariate in our analyses because IQ represents an inherent characteristic of our sample populations rather than a confounding variable we sought to remove. Furthermore, since IQ may not be causally related to motor ability, controlling for it would not enhance our ability to interpret the relationships between brain metabolites, sensory processing, and motor outcomes [46].

**Table 1:** Descriptive statistics.

	*Autism*				*TDC*			
	*N*	*M*	*SD*	*N*	*M*	*SD*	*ηp2*	*p*
Age	44	10.32	1.49	62	9.69	1.21	0.05	0.019
Sex (M:F)	42:2	-	-	46:16	-	-	-	< 0.001
Race (C: A: B: S)	35:5:4:0	-	-	48:2:5:5	-	-	-	0.106
Handedness (L:R)	4:40	-	-	5:57	-	-	-	1
ADOS								
Total	39	15.03	4.80	-	-	-	-	-
Social Interaction	39	8.31	2.54	-	-	-	-	-
Communication	39	3.67	1.59	-	-	-	-	-
RRSB	38	3.13	1.58	-	-	-	-	-
WISC-5								
GAI	40	104.5	17.98	48	112.98	11.17	0.08	0.008
Verbal Comprehension	41	101.34	16.50	49	111.92	11.69	0.13	< 0.001
Visual Spatial	41	109.54	18.63	49	113.18	12.41	0.01	0.271
Fluid Reasoning	41	105.68	17.68	49	110.35	11.34	0.03	0.134
Working Memory	41	99.59	16.98	49	110.80	11.89	0.13	< 0.001
Processing Speed	41	93.20	16.96	49	104.10	13.38	0.12	< 0.001
MABC-2								
Total	28	−2.69	2.20	45	1.67	3.01	0.38	< 0.001
Balance	28	−1.78	2.46	45	1.11	3.09	0.20	< 0.001
Manual Dexterity	28	−1.99	2.52	45	1.24	2.75	0.26	< 0.001
Aiming and Catching	28	−2.92	2.66	45	1.82	3.38	0.36	< 0.001
PANESS								
Total	26	9.44	12.67	60	−4.09	8.48	0.29	< 0.001
Timed	26	5.46	8.94	60	−2.37	6.18	0.21	< 0.001
Overflow	26	4.06	5.75	60	−1.76	5.08	0.21	< 0.001
Gaits-Stations	26	3.98	5.06	60	−1.73	4.40	0.25	< 0.001

*Race (C: A: B: S)* race (Caucasian: African American: Biracial: Asian), *RRSB* restricted, repetitive, stereotyped behavior.

### Group differences in motor ability

There were group differences on MABC-2 total scores (F(1, 71) = 43.84, p < 0.001, η_p_^2^ = 0.38), as well as on the balance (F(1, 71) = 17.51, p < 0.001, η_p_^2^ = 0.20), manual dexterity (F(1, 71) = 25.27, p < 0.001, η_p_^2^ = 0.26) and aiming and catching (F(1, 71) = 39.63, p < 0.001, η_p_^2^ = 0.36) subscale scores, with autistic children having lower scores than TDC for all measures. Group differences were also present for total PANESS scores (F(1, 84) = 33.81, p < 0.001, η_p_^2^ = 0.29), as well as on the timed (F(1, 84) = 21.97, p < 0.001, η_p_^2^ = 0.21), overflow (F(1, 84) = 22, p < 0.001, η_p_^2^ = 0.21) and gaits-stations (F(1, 84) = 27.84, p < 0.001, η_p_^2^ = 0.25) subscale scores, with the autism group having higher (worse) scores than the TDC group for all measures. In both the autism and TDC groups, there was a significant and negative correlation between MABC-2 total scores and PANESS total scores. See Supplementary Figs. 4 and 5.

### Sensory function is related to MRS GABA+ and Glx levels across autism and TDC groups together: evidence of path a

To ensure reproducibility of prior findings we first replicated correlations between sensory function and GABA+ and Glx levels in primary sensorimotor cortex and thalamus in the form of a heatmap (Supplementary Fig. 6). Supplementary Fig. 6 shows significant correlations between metabolite levels and both sensory reactivity and tactile perceptual sensitivity across groups, which provides further support for mediation analysis. When analysing these correlations across groups, sensorimotor (SM1) Glx levels were significantly related with SEQ Hypo-reactivity scores, SEQ Hyper-reactivity scores, sequential frequency discrimination (SQFD) and a proxy measure of lateral inhibition (LII; the difference between sequential and simultaneous discrimination). Thalamus GABA+ and Glx levels were significantly related to SEQ Seeking scores and simultaneous frequency discrimination respectively (SMFD). With the exception of the negative correlation between SM1 Glx levels with SQFD, all the remaining correlations were positive, indicating increased Glx and GABA levels were generally associated with increased sensory differences. These six significant relationships were subsequently used in mediation analyses, guiding the variables to choose to analyse the effect of independent variable (markers of E-I balance per region) onto the mediator (sensory function, path a).

### Sensory functioning scores are related to motor ability in both autism and TDC: evidence of path b

When analysing these correlations across groups, sensory reactivity as well as tactile perceptual sensitivity were related to motor ability (see Supplementary Fig. 7).

Tactile perception: Static detection threshold (SDT) scores were significantly related to *all* of the four MABC-2 measures and with three of the four PANESS (Total, Timed and Gaits-Stations) measures. Simultaneous amplitude discrimination thresholds (SMAD) scores were significantly related to three of four MABC-2 measures (Total, Balance, Aiming and Catching), and with two of PANESS measures (Total, Gaits-Stations). Sequential frequency discrimination threshold (SQFD) scores were significantly related to Total MABC-2 scores and Manual Dexterity subscale of MABC-2 scores. Simultaneous frequency discrimination threshold (SMFD) scores were significantly related to Balance subscale of MABC-2 scores. Finally, a measure of feedforward inhibition index (FFI, the difference between two detection threshold tasks) were significantly related to Gaits-Stations subscale of PANESS scores.

Sensory reactivity: SEQ Hyper- and Hypo-reactivity scores were significantly related to three of four MABC-2 measures (Total, Manual Dexterity, Aiming and Catching), and with *all* of the PANESS measures. SEQ Seeking scores were significantly related to Total MABC-2 scores, Aiming and Catching subscale of MABC-2 scores and Overflow subscale of PANESS scores.

Increased sensory scores were consistently correlated with lower MABC-2 scores and higher PANESS scores, indicating that greater sensory differences were associated with greater motor difficulties. These significant relationships were used later in the mediation effect analysis, guiding the variables to choose when analysing the mediation effect of mediator (sensory differences) onto dependent variable (motor control, path b).

### Relationship between sensory functioning scores and motor ability is generally group indifferent

**Fig. 2:**
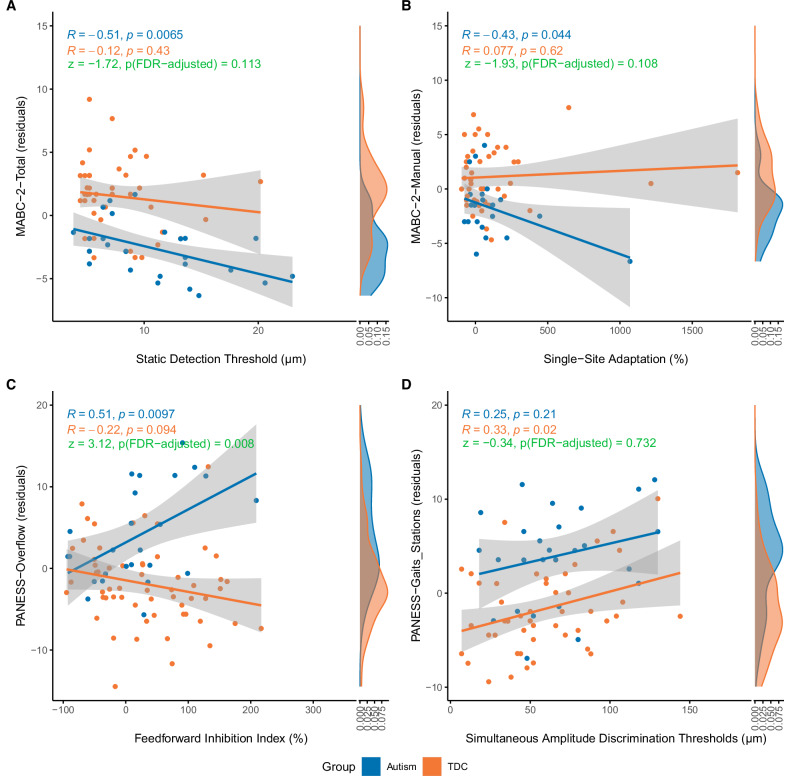
Correlations between motor ability and sensory functioning scores. The autism and TDC groups are represented as blue and orange respectively, distribution of motor ability for each group are also presented on the right side. **A** For the autism group, lower static detection threshold (i.e., better tactile perceptual sensitivity) correlated with higher MABC-2 total scores (i.e., better motor ability), a relationship that was otherwise absent in the TDC group. **B** For the autism group, lower single-site adaptation (i.e., better tactile perceptual sensitivity) correlated with higher MABC-2 Manual scores (i.e., better motor ability), a relationship that was otherwise absent in TDC. **C** For the autism group, lower feedforward inhibition index (i.e., worse tactile perceptual sensitivity) correlated with lower PANESS Overflow subscales (i.e., better motor ability). For TDC, lower feedforward inhibition index (i.e., worse tactile perceptual sensitivity) correlated with higher PANESS Overflow subscales (i.e., worse motor ability). **D** For TDC, lower simultaneous amplitude discrimination thresholds (i.e., better tactile perceptual sensitivity) correlated with lower PANESS Gaits-Stations (i.e., better motor ability), a relationship that was otherwise absent in the autism group. Values on the y-axis are negatives as values are residuals, due to controlling for sex and age as covariates. The results from the Fisher Z-Transformation are reported in green, with FDR correction applied to account for multiple comparisons.

We then showed that the relationship between sensory function and motor ability was generally indifferent across diagnostic groups. For brevity, we tested the significance of the difference between coefficients of correlation between autistic children and TDC children using Fisher Z-Transformation, on nine relationships which showed significant correlations between sensory function and motor ability in either one group or in both groups. We found only one relationship that showed statistically significant differences between the correlations across autism and TDC: the correlation between overflow subscale of PANESS scores and feedforward inhibition index (z = 3.12, p(FDR-adjusted) = 0.008; see Fig. 2).

### MRS GABA+ and Glx levels is unrelated to motor ability: no evidence of path c’

As hypothesized, motor ability was generally not associated with brain metabolite levels (p > 0.05, see Supplementary Fig. 8). The general insignificance of these correlations suggests there is no total effect of GABA+ and Glx levels in SM1 and thalamus on motor control; this effect might be better reflected by a cascade which takes sensory function as the first layer that is influenced by brain metabolite levels and subsequently leads to changes in motor ability. We discuss this further below.

### The mediation effect of sensory reactivity on the relationship between motor ability, GABA+ and Glx levels

Given the significant relationships observed between brain metabolite levels and sensory scores, and between sensory scores and motor ability, we then performed mediation analysis between brain metabolite levels, sensory scores and motor ability to assess whether there is an indirect effect as mediated by differences in sensory scores. We identified nine combinations where sensory functioning scores significantly mediated the relationship between brain metabolite levels and motor ability.

**Fig. 3:**
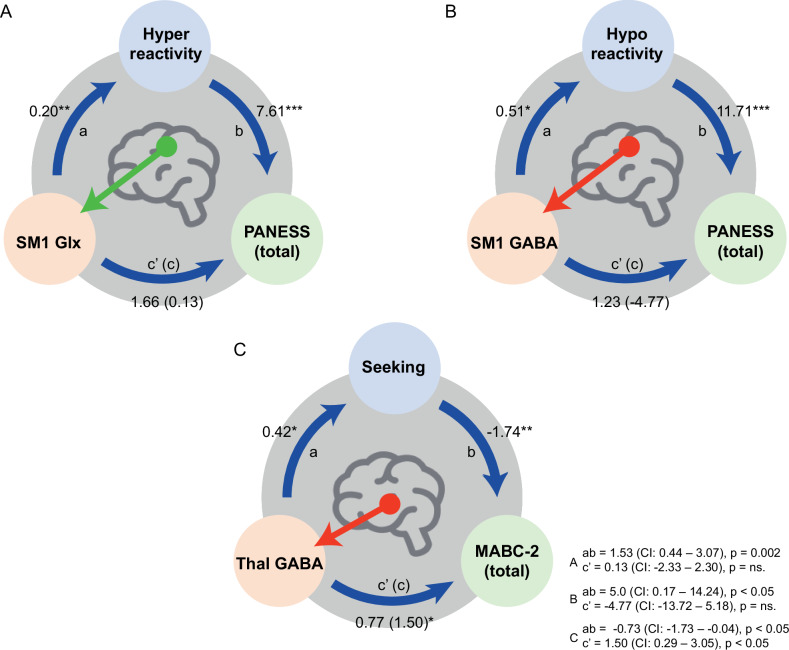
Mediation effects of sensory reactivity scores on correlations between motor ability and brain metabolite levels. The beta coefficients and significance of associations between brain metabolite levels, sensory reactivity scores and motor ability scores are shown in the figures. **A** The mediation effect through SEQ Hyper-reactivity Score from SM1 Glx. **B** The mediation effect through SEQ Hypo-reactivity Score from SM1 GABA. **C** The mediation effect through SEQ Seeking Score from Thal GABA. Excitation are coloured green and inhibition are coloured red. The indirect effect (ab) indicates the effect of exposure on the outcome that works through the mediator (sensory reactivity scores), while the direct effect (c’) indicates the effect of metabolite levels on the motor ability scores absent the mediator. Significance is indicated by “*”. * = p < 0.05, ** = p < 0.01 and *** = p < 0.001.

**Fig. 4:**
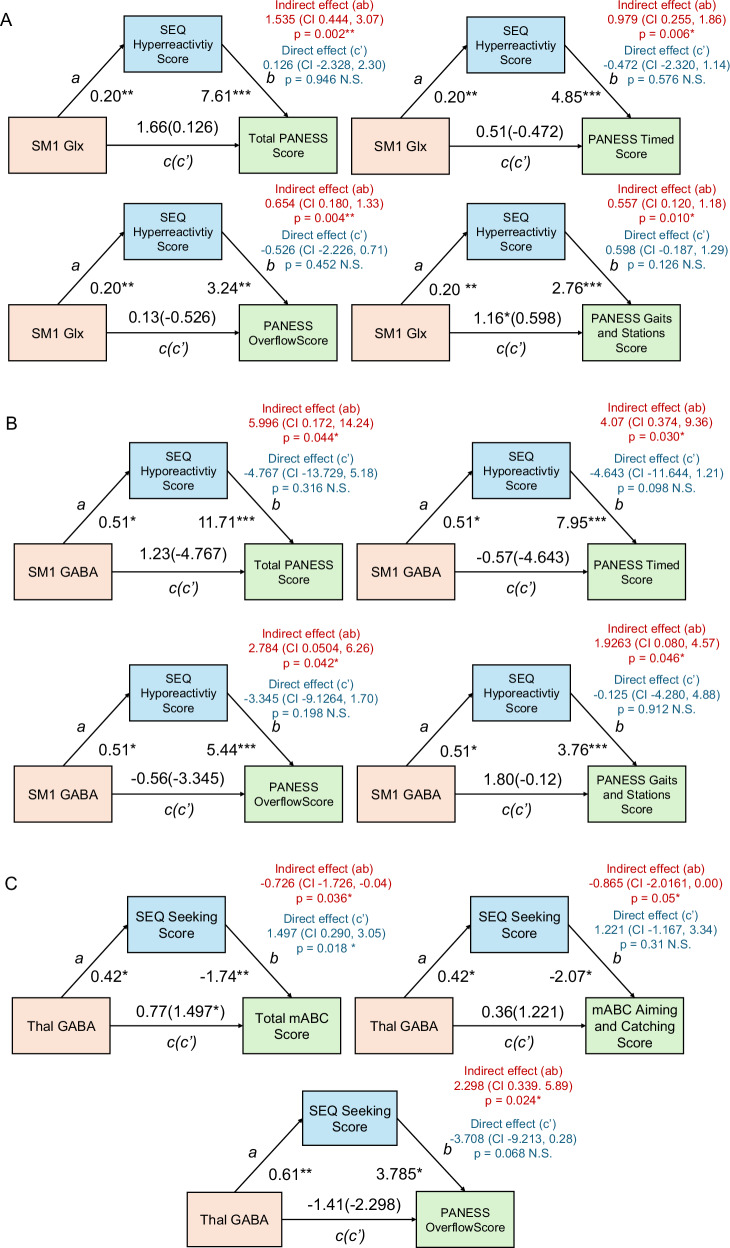
Full details of the mediation effect. **A** SEQ Hyperreactivity Scores fully mediated the relationships between SM1 Glx levels and all of the PANESS score. **B** SEQ Hyporeactivity Scores fully mediated the relationships between SM1 GABA levels and all of the PANESS scores. **C** SEQ Seeking Scores fully mediated the relationships between thalamic GABA levels and MABC-2 Aiming and Catching Score scores and PANESS Overflow scores, partially mediated the relationships between thalamic GABA levels and total MABC-2 score. The indirect effect (ab) indicates the effect of exposure on the outcome that works through the mediator (sensory reactivity scores), while the direct effect (c’) indicates the effect of metabolite levels on the motor ability scores absent the mediator. Significance is indicated by “*”. * = p < 0.05, ** = p < 0.01 and *** = p < 0.001.

These mediation effects showed surprising consistency; only sensory reactivity play the role of mediator. Also, sensory reactivity scores each fully mediated the effect from one specific region-specific metabolite, which means when considering the indirect effect from mediator, the direct effect from the independent variables to dependent variables is not significant. These are detailed in Fig. 3. SEQ Hyper-reactivity Scores fully mediated the relationships between SM1 Glx levels and PANESS total score (see Fig. 3A). SEQ Hypo-reactivity Scores fully mediated the relationships between SM1 GABA levels and PANESS total score (see Fig. 3B). In contrast, SEQ Seeking Scores partially mediated the relationships between thalamic GABA levels and MABC-2 total score (see Fig. 3C). See Fig. 4 for detail.

We then conducted a sensitivity analysis for the casual mediation analyses that showed significance. Across all the mediation models, the absolute value of *rho* ranged from 0.40–0.50 (see Supplementary Table 2), indicating a relatively consistent and moderate degree of robustness of our model in comparison with the sensitivity analyses conducted in other statistical frameworks [47, 48].

Although the prerequisite correlations for mediation analysis were present within the autism group (sensory function scores significantly correlated with both metabolite levels and motor abilities), the mediation models did not show statistically significant results. We attribute this finding to insufficient statistical power due to the reduced sample size in individual group analyses (see Discussion section).

## Discussion

Here, we tested a hypothetical mediation model to assess the highly prevalent co-occurrence of sensory and motor dysfunction in autistic children. We focused on sensory and motor features that are characteristic of autism but may also occur in neurotypical populations. Our analysis examined both overall patterns across groups and group-specific relationships to understand whether the underlying mechanisms contributing to autism aetiology operate similarly across populations. Understanding these relationship patterns and their consistency or variation between autism and TDC groups provides insight into the etiological mechanisms underlying autism-related traits.

We predicted a significant association between measures of sensory and motor function and that autistic patterns of sensory-motor atypicality would be driven by differences in MRS markers of excitation and inhibition balance. Consistent with our previous findings, we found an association between atypical sensory function and brain GABA+ and glutamate+glutamine levels [10]. Having replicated these findings, we then examined how these sensory differences are associated with autistic patterns of motor difficulty as assessed using two observational measures: PANESS and MABC-2. Consistent with several prior studies [48, 49], we found autistic children show significant, broad-based difficulties on these two measures. Consistent with our hypotheses, we found that these motor difficulties were associated with sensory atypicalities at the tactile perceptual and the broader sensory reactivity level, adding further support to the theory that sensory differences contribute to motor difficulties. Further, as predicted, we did not observe significant correlations between MRS measure of GABA+ and Glx levels and motor function; rather, our results showed that differences in motor function could be explained by differences in GABA+ and Glx levels *indirectly*, with mediating sensory difficulties. These findings suggest that the sensory and motor difficulties observed in autistic children might be intricately linked. Additionally, they suggest that differences in brain GABA+ and Glx levels might directly contribute to sensory differences and that distinct sensory profiles may differentially mediate metabolite-motor associations.

We and others have previously established sensory differences in autistic children [50] both in terms of tactile perception [17] and sensory reactivity [10]. Our previous and current findings suggest that tactile perceptual sensitivity and sensory reactivity are associated with elevated excitation [10] or reduced inhibition [51], with this E-I imbalance leading to changes in endogenous neural noise, which can then lead to changes in sensory mechanisms such as sensory gating and lateral inhibition [52]. Building on these findings, here we demonstrate more nuanced and detailed relationships between our markers of E-I balance (GABA+ and Glx levels) and sensory function, as detailed below.

Motor difficulties in autism are generally well established. Here we used a pair of complimentary motor assessments (i.e., the MABC-2 and PANESS) to measure motor difficulties in autistic children. The MABC-2 includes tasks covering manual dexterity (fine motor control with hands), aiming and catching (catching and throwing skills), and balance. Subscales for each task category represent performance within that specific domain. The PANESS includes tasks covering gaits (walking), stationary movements (primarily hand and foot movements), and timed movements (frequency-related tasks). The summary subscales represent different motor abilities: The Overflow subscale quantifies excess movements that appear during the Gaits and Stations tasks as well as the Timed tasks; the Gaits and Stations subscale represents axial abilities (balance, coordination, and postural stability), as well as overflow and involuntary movements during these tasks; the Timed subscale represents speed, overflow, and dysrhythmia in the timed tasks; the Total PANESS score is the sum of the Gaits and Stations and Timed subscales, representing overall motor performance across the entire motor assessment. In line with previous work [48, 49, 53], our results showed that motor ability was poorer in autism compared to TDC for both assessments, providing validity for these two measures in assessing autism-associated motor difficulties. Group differences were observable for both total and subscale scores of both measures, suggesting general rather than specific motor difficulties.

We subsequently tested whether differences in sensory function were correlated with motor ability in both groups combined. These correlations between sensory function and motor ability were generally suggesting that more difficulty with sensory function predict worse motor performance. Our findings that sensory reactivity and static detection thresholds form significant correlations with a wide range of motor abilities suggests that sensory processing and modulation are broadly associated with motor development rather than contributing to specific motor patterns in childhood.

In contrast, correlations between certain tactile perceptual measures and motor ability were not consistently generalizable across groups when considering diagnosis. We found that the correlation between FFI and the ‘overflow’ subscale scores of the PANESS showed a significant difference between groups (See Fig. 2). Specifically, higher FFI scores (indicating reduced feedforward inhibition) were significantly correlated with increased motor overflow index in the autism group, while they were significantly correlated with decreased motor overflow index in the TDC group. The FFI was a subscale derived from the difference between static and dynamic detection threshold tasks in the vibrotactile psychophysical assessment. Our previous study suggested that the detection difference between dynamic increases in vibration intensity and static vibration intensity tasks results from feedforward inhibition mechanisms activated by the sub-threshold vibration present in the dynamic detection task [54]. Importantly, FFI-related cortical interneurons receive direct thalamic input [55] and FFI in the sensorimotor system may also amplify cerebellar signals [55]. Our findings suggest that differences in feed-forward inhibition in autism may drive difficulties in both sensory and motor function by affecting the sharpening of tuning in thalamocortical network to sensory stimuli and motor execution [56].

We were unable to demonstrate a direct relationship between MRS markers of E-I balance (GABA+ and Glx levels) and motor ability across most measures. However, we did identify one significant correlation between thalamic Glx and the PANESS timed subscale specifically within the autism group, where higher thalamic Glx levels were associated with better performance on timed motor tasks involving movement speed and accuracy. We suggest this finding may reflect the critical role of the thalamus in signal integration, where elevated glutamatergic signalling in certain neurodevelopmental conditions could enhance specific motor control processes, thereby improving performance on particular aspects of motor function [57]. This finding aligns with the E-I balance hypothesis for autism, as the elevated thalamic excitation reflected by higher thalamic Glx levels may contribute to this correlation being significant in the autism group rather than the TDC group.

Instead, we found significant mediation effects with sensory reactivity as the mediator. The mediation effects broadly suggest that differences in GABA+ and Glx levels contribute to sensory difficulties with subsequent impact on motor function. However, the nuanced findings shed more light on the intricate relationship between these measures. Findings that different sensory experience metrics each mediated an effect from one region-specific metabolite suggest different roles and mechanistic underpinnings of sensory behaviours, despite hyper- hypo-reactivity and seeking often co-occurring within the same individual [17].

The dissociation between hyper-reactivity, hypo-reactivity and seeking in mediation effect, and the correspondence of the brain regions related to sensory and motor differences, support our view that E-I balance are perhaps useful umbrella terms but that a more granular view, i.e. understanding that different processes and brain regions have different roles in driving autistic behaviours, is key in moving the field forward in such a manner that we can have a more detailed understanding of the mechanisms that drive these behaviours. The finding that associations between motor ability and metabolite levels were regionally specific rather than metabolite specific, suggests that metabolite origins are more important in motor control. Earlier studies suggest that Glx and GABA+ levels might have opposite effects on motor behaviours [26, 58–60] whereas our finding suggests that the overall effect of the excitation and inhibition balance in the neural system is the key factor, but are regionally specific, and thus, differentially impact motor ability. This makes sense given the differentiation of brain regions for suiting operation and control of sensory and motor functions.

Similarly, “sensory differences” is also an umbrella terms, but our work shows that its biological drivers depend on the type of behaviour. We have expanded on the need for a more granular approach in our recent framework proposal [50]. Our finding that most of the mediation effects are full mediation, also supporting the idea that sensory differences are a first-order consequence of altered excitation-inhibition, and motor differences are a second-order consequences [61, 62].

Our prior published work suggests that both GABA and glutamate are related, and that hyper-reactivity, hypo-reactivity and seeking are positively related [10], and it is difficult in the current sample to covary for these relationships. However, mediation sensitivity analysis suggests a moderate robustness of our hypothetic model, although the lack of sensitivity analysis in similar studies prohibits the comparison of our model. However, the small range of absolute values of sensitivity parameters across different mediation effects still suggests a consistent and robust mediation model within our study.

Taken together, our findings support a hypothetical model for the sensory and motor difficulties in autism. Excitation and inhibition imbalance caused by changes in Glu and GABA affects how an individual may respond to sensory input, which in turn prohibits the accurate guidance of motor behaviours, by influencing top-down motor control.

It has been suggested that the autism-associated differences of motor planning, may be, at least in part driven by differences of low-level sensory processing. It has also been suggested that differences of low-level sensory processing are driven by alterations of E-I balance. Based on that we hypothesised significant mediation effects with tactile perception as the mediator, but we failed to find such an effect. This is surprising given that motor control utilizes the somatosensory system, and our MRS voxel encompassed sensorimotor cortex. However, our tactile measures may be too specific to relate to more complex motor behaviours measured here, and moreover, the motor control measures here do not solely reflect primary motor processing but rather higher-order motor planning, likely involving premotor regions.

While our results broadly support our hypothetical model, we are certainly not able to demonstrate causation. Given the nature of these relationships, establishing causation will be inherently difficult in a cross-sectional sample. For instance, it is difficult to determine whether sensory differences precede motor differences given that individual differences in motor ability are difficult to determine at the age when sensory differences become apparent (i.e., as early as 6 months of age). Nonetheless, future studies attempting to test the same or similar hypothetical models should find new ways to establish causation e.g. through shiftability design studies [63] or TMS [64]. While this might be difficult, establishing causation between these constructs will substantially contribute to our understanding of the aetiology of autism and inform diagnosis, intervention, and therapy. While we managed to include 44 autism participants and 62 TDC participants, a larger sample will allow us to perform more complex structural equation modelling to look into these relationships in more complex detail.

As a study aiming to investigate the biology of autism, we failed to find significant mediations in the autism group alone. We rather found most of these relations were only significant when including both autism and TDC groups, which suggests that our findings are more general rather than autism specific. However, our findings do provide insight into understanding atypical sensory and motor behaviours in autism; First, we see more severe sensory and motor difficulties in autism, as well as higher Glx, and thus, rather than a group difference, our findings suggest this may simply be an association of traits along a dimensional scale. Second, the absence of clear mediation specific to autism may simply be due to insufficient sample size as we found significance of the correlations and mediation effects when including both groups, but not in the separate groups. We also note that indeed, for mediation analyses, larger sample sizes are required [65]. Future studies with larger sample sizes preregistered to identify these associations, may have sufficient power to detect mediation effects within individual diagnostic groups and clarify whether these neurobiological pathways are autism-specific or represent broader neurodevelopmental mechanisms. Of course, it would be of significant interest to separate Glutamate and glutamine to provide a more detailed look at glutamate alone, as both a neurotransmitter and involvement in energy metabolism. However, dedicated sequences (e.g. TE-averaging [66]) would be required, or imaging at higher magnetic fields, which would be of strong interest in the future. We should also note that we have a surprisingly small sample of autistic girls, despite a reported 4:1 ratio in the literature [67]. We therefore cannot generalize these findings to autistic girls, however, there is limited evidence that E-I balance [23], sensory [68] and motor atypicalities differ between boys and girls [69].

## Supplementary information

SUPPLEMENTAL MATERIAL

## Data Availability

The code supporting the findings of this study is publicly available on the Open Science Framework (OSF) at https://osf.io/5unmc/overview. The repository includes scripts for reproducing the figures and all associated output figures. Due to General Data Protection Regulation (GDPR) and data transfer agreements, the datasets analysed during the current study are not publicly available but can be requested from CNIR (Mostofsky) through standard project and data access procedures.
