# Supplementary material for: Distinct sensory atypicalities bridge the gap between brain chemistry and motor dysfunction in autism
Source: Transl Psychiatry. 2026 May 8;16:320. doi: 10.1038/s41398-026-04036-z (PMC13324517; doi:10.1038/s41398-026-04036-z)
Supplement: Supplementary file 1 — SUPPLEMENTAL MATERIAL [file 41398_2026_4036_MOESM1_ESM.docx]

**Supplemental Materials: Distinct Sensory Atypicalities Bridge Brain Chemistry and Motor Dysfunction in Autism**

**
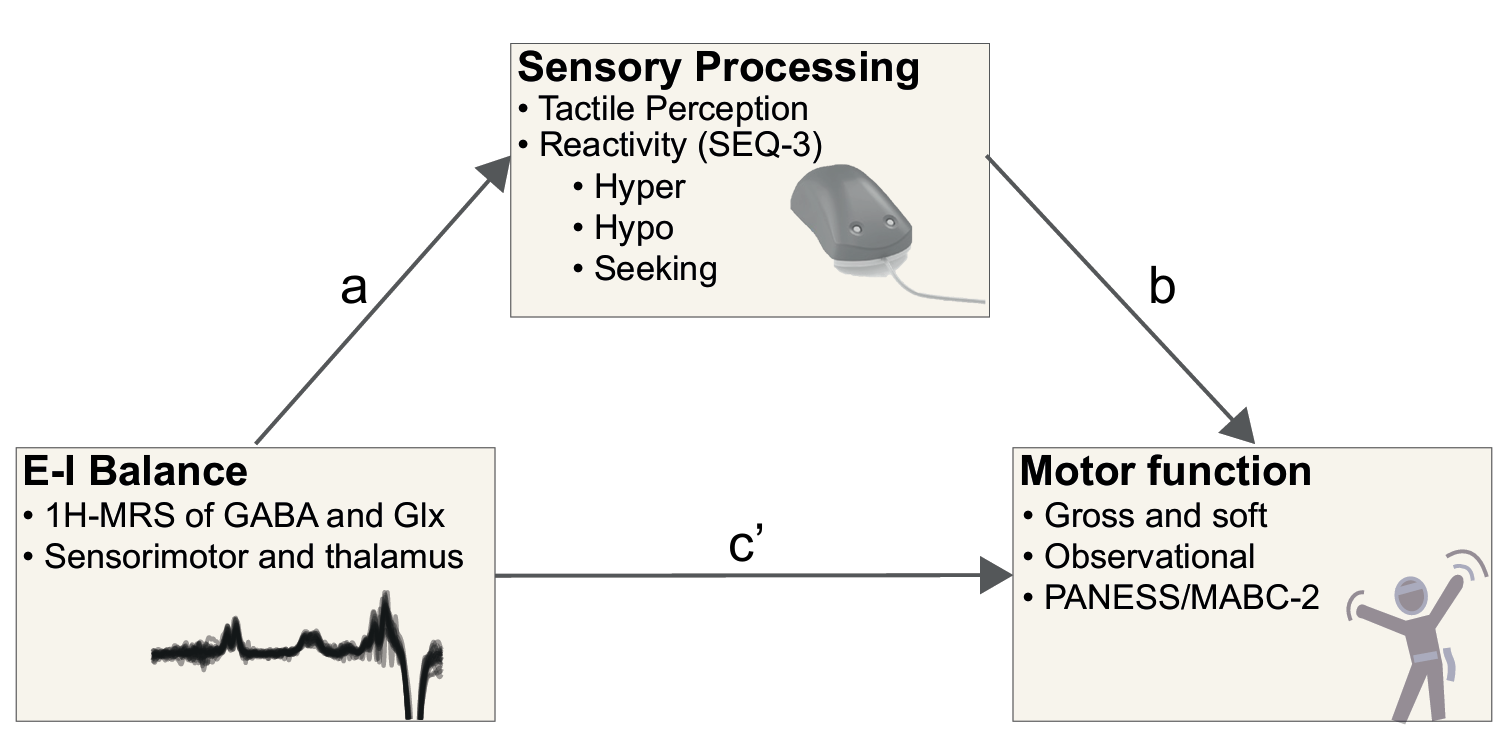
**

**Supplementary Figure 1.** **Hypothetical mediation model**. In this hypothetical model, E-I balance drives differences in sensory function, which in turn drives differences in motor ability. We are hypothesizing that there is either a direct effect of MRS markers of E-I balance on motor ability, or that the effect of E-I markers on motor ability is mediated by sensory function. Path a has already been tested and reported on in He et al., (2021). Higher SM1 and Thal Glx levels predict worse tactile perceptual sensitivity. Paths b and c’ remain untested. Our hypotheses suggests that paths a and b will be significant, but that path c’ may not. In our analyses, we operationalised E-I balance as glutamate + glutamine (Glx) and GABA + macromolecules (GABA+) levels, sensory processing as sensory experience questionnaire scores and tactile perception, motor function as performance on the MABC-2 and PANESS.

**Supplemental Methods**

***Tactile Perceptual Sensitivity task.***


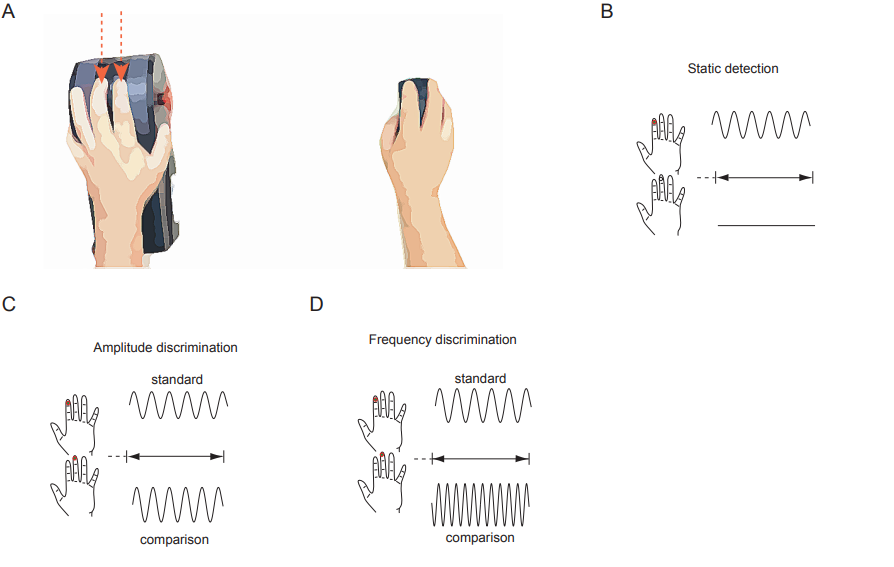


**Supplementary Figure 2. Visual schematic for tactile perception tasks. a.** Visualization of the vibrotactile psychophysical task. Participants were asked to put their left digit 2 and left digit 3 on the vibrotactile machine, stimuli were delivered with 5 mm probes. The right hand of the participants were used to give real-time feedback to the tasks. **b.** For static detection task, participants detected which digit received the stimulation with a fixed frequency. **c.** For amplitude discrimination task, participants compared the amplitude of stimulation they received across the digits. **d.** For frequency discrimination task, participants compared the frequency of stimulation they received across the digits.

**Comprehensive detailing of MRS data acquisition, pre-processing, modelling and quantification.**

***Acquisition.***

During the acquisition period, the MRI scanner underwent hardware and software upgrades. MRS protocols also went through improvement to address drift and frequency offsets. The different parameters for each phase were (as detailed in (1) and on https://osf.io/xhqwu/):

- **Phase 1:** Macromolecule-suppressed GABA-edited MEGA-PRESS (February 2014 - April 2015); 20-ms editing pulses applied alternated at 1.9 ppm (GABA-ON) and 1.5 ppm (GABA-OFF) with a separate water-unsuppressed reference scan (TE = 80 ms) after the water-suppressed scan.
- **Phase 2:** Macromolecule-suppressed GABA-edited MEGA-PRESS (August 2016 - July 2018) with interleaved water referencing and a crusher-gradient lock; with 20-ms editing pulses applied alternated at 1.9 ppm (GABA-ON) and 1.5 ppm (GABA-OFF); interleaved water-unsuppressed reference scan (TE = 80 ms) and dynamic scanner frequency update every 20 averages; the crusher-gradient lock was applied to prevent a rotation-dependent eddy-current-induced editing pulse frequency offset.
- **Phase 3:** HERMES for simultaneous editing of GABA+macromolecules and GSH (July 2018 - December 2019). 20-ms editing pulses applied in a four-step scheme at (A) 1.9 ppm & 4.56 ppm, (B) 1.9 ppm, (C) 4.56 ppm, (D) no editing pulse; interleaved water-unsuppressed reference scan (TE = 80 ms) and dynamic scanner frequency update every 20 averages.

***Pre-processing, modelling, and quantification.*** MRS data were processed using Gannet 3.1. Preprocessing: Spectral preprocessing included 3-Hz exponential line broadening, zero-filling to 32768 points, frequency-and-phase correction of individual averages using the spectral registration method, averaging of OFF and ON spectra and subsequent subtraction to obtain GABA-edited (and, for HERMES, GSH-edited) difference spectra.

GannetFit was then used to model the difference spectra, edit-OFF spectra, and water-unsuppressed spectra. For GABA-edited difference spectra, a combined GABA-Glx model (2.79 ppm and 4.1 ppm) was used to fit the 3 ppm GABA and the 3.75-ppm Glx resonances simultaneously using a single Gaussian model for the GABA peak (between 2.79 ppm and 3.2 ppm), a double-Gaussian model for the Glx doublet (between 3.4 ppm and 4.1 ppm) and baseline terms to account for baseline distortions caused by water or lipid signal residuals. For edit-OFF spectra, a double-Lorentzian model was used to fit the singlets from total creatine (at 3.02 ppm) and total choline (3.20 ppm) between 2.6 ppm and 3.6 ppm, using a fixed peak separation (0.18 ppm), a zero-order phase term, and a linear baseline term. For unsuppressed water spectra, a single mixed Lorentzian-Gaussian model was used to fit between 3.8 ppm and 5.6 ppm, including a linear baseline term and a vertical offset.

GannetCoregister and GannetSegment modules were used to co-register voxel to the T1-weighted anatomical image from the same acquisition. Fractional tissue volumes for grey matter (GM), white matter (WM), and cerebrospinal fluid (CSF) were determined for each voxel using tissue segmentation implemented in SPM12 as part of the Gannet module. Quantitative estimates for each metabolite were derived from the model areas for the different metabolites and the segmentation results, an alpha-corrected concentration estimate for GABA: *GABAAlphaCorr*, was derived by accounting for different amount of GABA between grey and white matter, assuming a 2:1 ratio (i.e., GABA levels in grey matter are twice as high as in white matter). We note that while this ratio is not known exactly, it is based on estimated ratios and suggested that accounting for an alpha is more appropriate than not including the alpha component (2). The final quantitative metabolite estimates were then used in the statistical analysis. Taking metabolite estimates relative to tissue-water into account helps establish that potential group effects and correlations are indeed driven by individual differences in the nominator (GABA or Glx, respectively), rather than in the denominator (water).

The GannetLoad and GannetFit output plots for each dataset were visually inspected by a member of the study team (GO, ~9 years of experience using edited MRS). Data themselves or the fits considered unusable. subtraction artefacts and lipid contamination in cortical voxels, were excluded from further analyses.

***Directionality exploration of mediation analysis.***

We focused our mediation analysis on sensory functioning scores as mediators between metabolite levels and motor abilities, based on previous studies demonstrating the impact of sensory input on motor performance (3-5). However, we also tested the opposite direction, whereby motor performance acts as the mediator between metabolite levels and sensory outcomes.

For analysis of the reverse direction, we observed limited significant correlations between motor abilities and metabolite levels (only thalamic Glx levels correlated with PANESS Timed subscores in the autism group). Since mediation analysis requires significant correlations between the mediator and both independent and dependent variables, we could only construct a limited number of alternative mediation models. Specifically, we tested alternative mediation models within the autism group using FFI and SMFD (sensory measures), thalamic Glx levels, and PANESS Timed subscores in different configurations: Model 1 examined whether thalamic Glx mediates the relationship from sensory processing (FFI or SMFD) to motor performance (PANESS Timed subscores), while Model 2 tested whether thalamic Glx mediates the relationship from motor performance (PANESS Timed subscores) to sensory processing (FFI or SMFD). However, these alternative mediation models yielded non-significant results and lack direct theoretical support in the existing literature, reinforcing our original hypothesis that sensory processing mediates the relationship between brain metabolites and motor abilities.

**Supplemental Results**

**
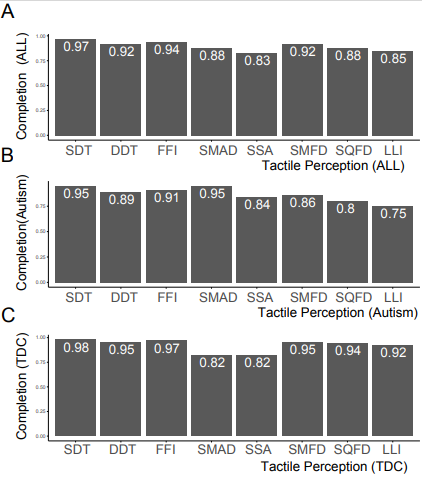
**

**Supplementary Figure 3. Tactile perception tasks completion rates.** There are generally high completion rates across all tactile perception tasks both when ignoring the diagnosis and in separate groups.

χ^2^ tests were performed to compare completion rates between autism and TDC groups, with significant differences found for SQFD (χ^2^= 4.076, p = 0.044) and LLI (χ^2^ = 4.818, p = 0.028), likely reflecting more difficulty for these particular measures.

**Comparisons of motor assessment between group**

We compared motor ability between autistic and TD children using linear models with total or subscale scores from the MABC-2 or PANESS as the dependent variable, age and sex as covariates, and diagnosis (autism or TDC) as the independent variable.


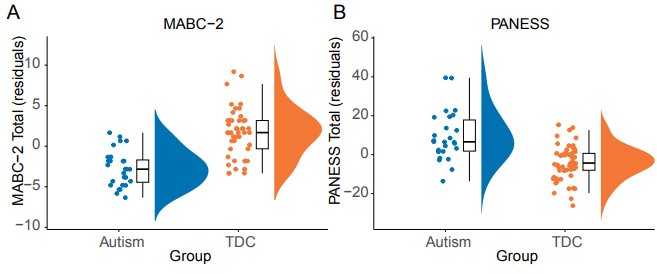


**Supplementary Figure 4. Visualisation of the comparisons on MABC-2 and PANESS total scores.** The autism and TDC groups are represented as blue and orange circles respectively. **a.** Higher scores on the MABC-2 indicates better motor ability. **b.** Higher scores on the PANESS indicates poorer motor performance. As can be seen in both panels, the autism group substantially differed from the TDC group, showing more difficulties with motor control on both the MABC-2 and PANESS. Values on the y-axis are negatives as values are residuals, due to controlling for sex as a covariate.

**Correlations of two motor assessment**


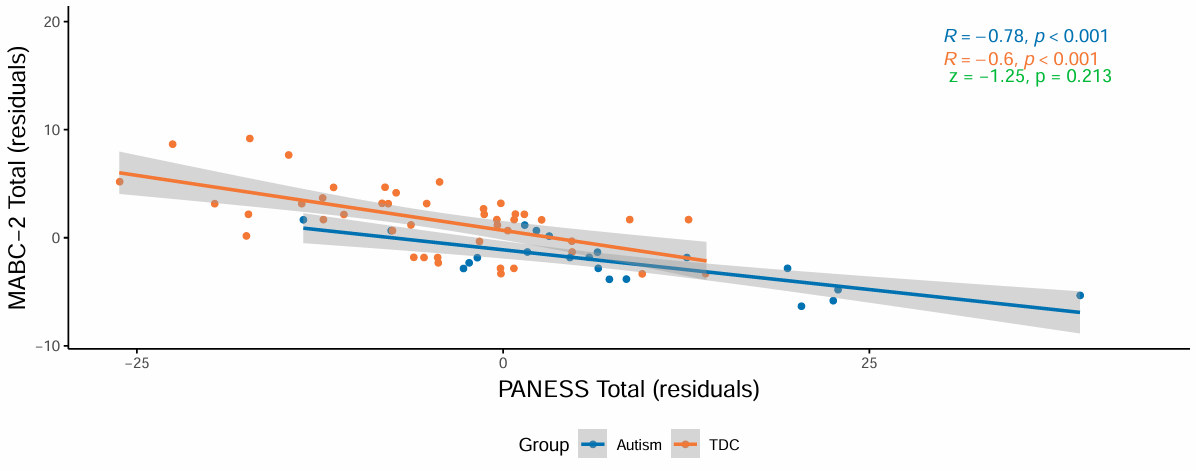


**Supplementary Figure 5. Visualisation of the correlations between MABC-2 and PANESS total scores.** Regression plots for autism (blue circles) and TDC (orange circles). Higher scores on the PANESS indicates poorer fine and gross motor ability, while lower MABC-2 scores indicates greater motor impairment, the negative correlations show that poorer fine and gross motor skills are correlated with greater motor impairment.

**Correlations between MRS GABA+ and Glx levels and sensory functioning scores**


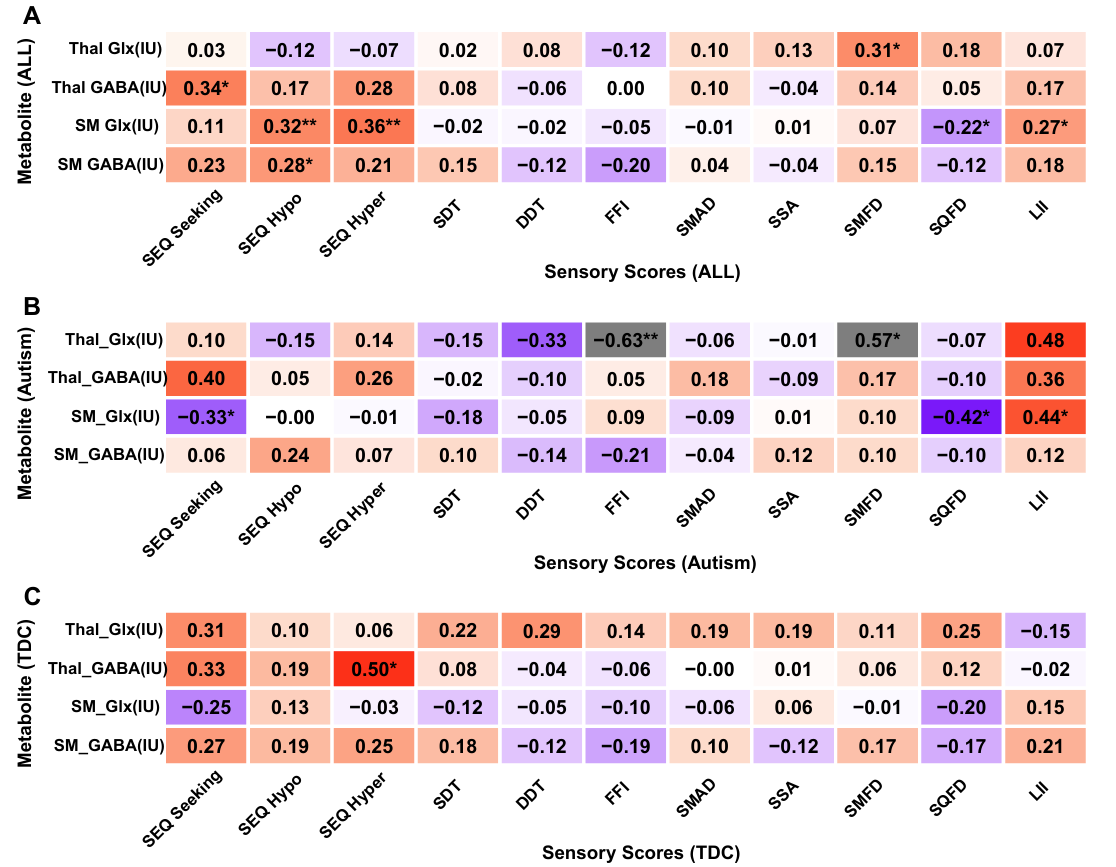


**Supplementary Figure 6. Heatmap of correlations between brain metabolite levels and sensory function scores.** Glx and GABA+ levels in thalamus and sensorimotor cortex are presented on y-axis. Sensory experience questionnaire scores and performance indices from the vibrotactile battery are presented on the x-axis. Positive correlations are coloured orange and negative correlations are coloured purple. Significance is indicated by “*”. As can be discerned, SEQ Seeking, SEQ Hypo, SEQ Hyper, SMFD, SQFD and LLI were the sensory functioning scores correlated with metabolite levels. * = p < 0.05, ** = p < 0.01 and *** = p < 0.001. SDT = Static detection threshold, DDT = Dynamic detection threshold, FFI = feedforward inhibition index, SMAD = simultaneous amplitude discrimination threshold, SSA = single-site adaptation, SMFD = simultaneous frequency discrimination threshold, SQFD = sequential frequency discrimination threshold, LII = lateral inhibition index.

**Correlations between motor ability and sensory functioning scores**


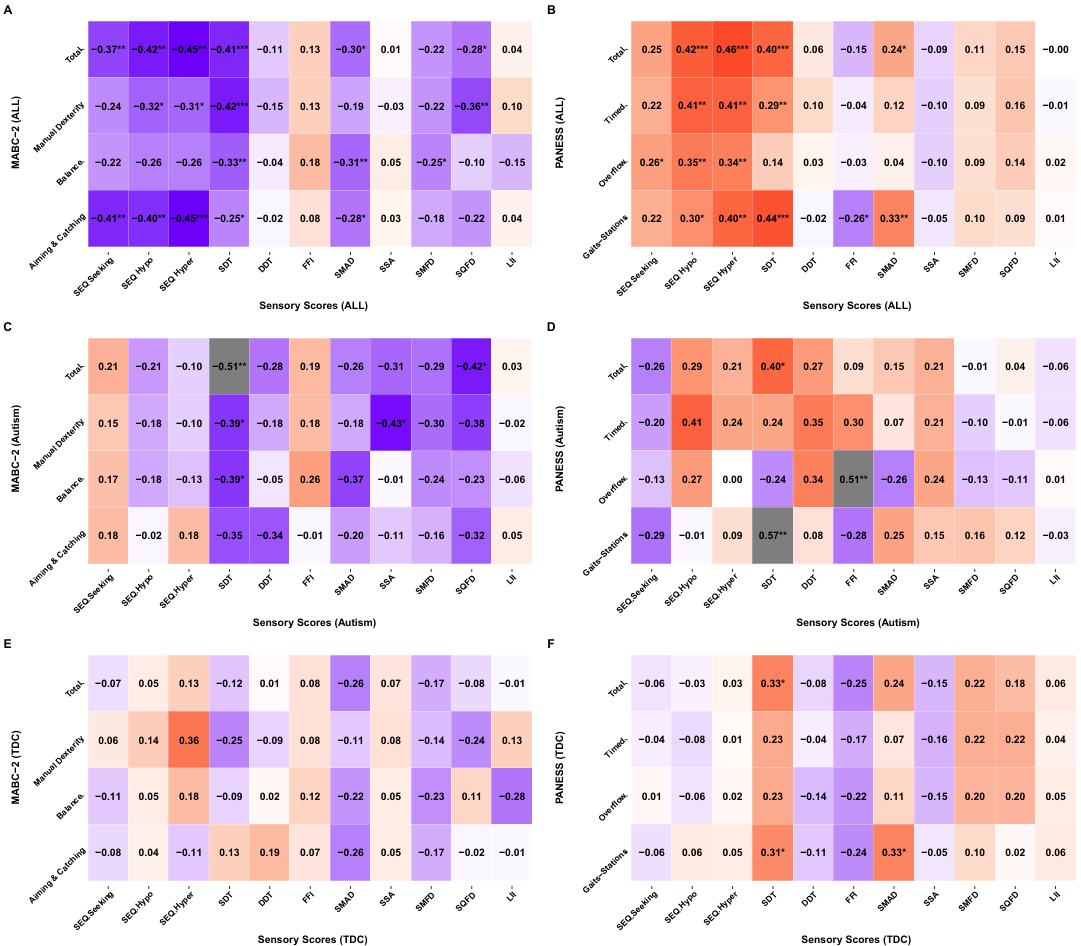


**Supplementary Figure 7. Heatmap of correlations between motor ability and sensory functioning scores.** MABC-2 and PANESS total and subscale scores are presented on y-axis. Sensory experience questionnaire scores and performance indices from the vibrotactile battery are presented on the x-axis. Positive correlations are coloured orange and negative correlations are coloured purple. Significance is indicated by “*”. As can be discerned, SEQ Seeking, SEQ Hypo, SEQ Hyper, statistic detection thresholds and simultaneous amplitude discrimination thresholds were the sensory functioning scores indices most correlated with motor ability. Note that higher scores on better motor ability on the MABC-2 but worse motor ability on the PANESS. When taken together, these correlations strongly suggest that less sensory experience symptoms and better tactile perceptual sensitivity are associated with better motor ability and vice versa. * = p < 0.05, ** = p < 0.01 and *** = p < 0.001. SDT = Static detection threshold, DDT = Dynamic detection threshold, FFI = feedforward inhibition index, SMAD = simultaneous amplitude discrimination threshold, SSA = single-site adaptation, SMFD = simultaneous frequency discrimination threshold, SQFD = sequential frequency discrimination threshold, LII = lateral inhibition index.

**Correlations between MRS GABA+ and Glx levels and motor ability**


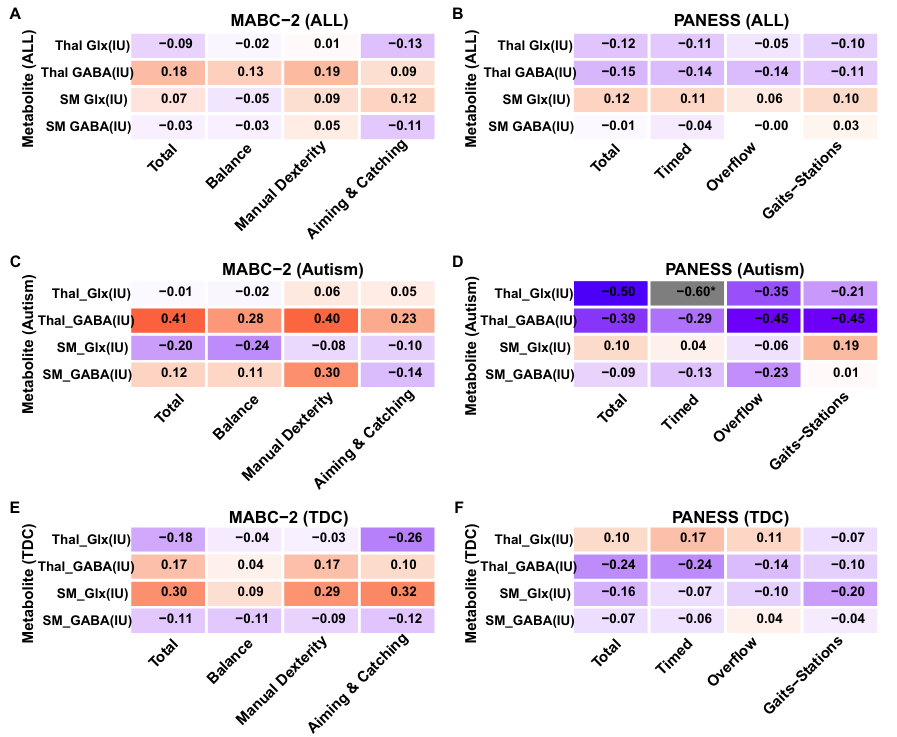


**Supplementary Figure 8. Heatmap of correlations between motor ability and metabolite levels.** MABC-2 and PANESS total and subscale scores are presented on y-axis. Glx and GABA+ levels in SM1 and thalamus are presented on the x-axis. Positive correlations are coloured red and negative correlations are coloured blue. Significance is indicated by “*”. As can be discerned, there is no significant correlation between motor ability and metabolite levels. Note that higher scores reflect better motor ability on the MABC-2 but worse motor ability on the PANESS. * = p < 0.05, ** = p < 0.01 and *** = p < 0.001.

**Tables**

**Supplementary Table 1. Completion rates for MRS, sensory and motor assessment**

|  | *Autism* | | | | | | TDC | |  |
| --- | --- | --- | --- | --- | --- | --- | --- | --- | --- |
|  | *N* | *Rate* | |  | *N* | | | *Rate* | |
| Total | 44 | - | |  | 62 | | | - | |
| MRS |  |  |  | | |  | |  |  |
| SM_Glx | 44 | 100% | |  | 59 | | | 95.2% | |
| SM_GABA | 44 | 100% | |  | 59 | | | 95.2% | |
| Thal_Glx  Thal_GABA | 29  29 | 65.9%  65.9% | |  | 33  33 | | | 53.2%  53.2% | |
| SEQ |  |  | |  |  | | |  | |
| SEQ_Seeking | 37 | 84.1% | |  | 40 | | | 64.5% | |
| SEQ.Hypo | 37 | 84.1% | |  | 40 | | | 64.5% | |
| SEQ.Hyper | 37 | 84.1% | |  | 40 | | | 64.5% | |
| Vibrotactile |  |  | |  |  | | |  | |
| SDT | 42 | 95.5% | |  | 61 | | | 98.4% | |
| DDT | 39 | 88.6% | |  | 59 | | | 95.2% | |
| FFI | 40 | 90.9% | |  | 60 | | | 96.8% | |
| SMAD  SSA  SMFD  SQFD | 42  37  38  35 | 95.5%  84.1%  86.4%  79.5% | |  | 51  51  59  58 | | | 82.3%  82.3%  95.2%  93.5% | |
| LII | 33 | 75.0% | |  | 57 | | | 91.9% | |
| MABC-2 |  |  | |  |  | | |  | |
| Total | 28 | 63.6% | |  | 45 | | | 72.6% | |
| Balance | 28 | 63.6% | |  | 45 | | | 72.6% | |
| Manual Dexterity | 28 | 63.6% | |  | 45 | | | 72.6% | |
| Aiming and Catching | 28 | 63.6% | |  | 45 | | | 72.6% | |
| PANESS |  |  | |  |  | | |  | |
| Total | 26 | 59.1% | |  | 60 | | | 96.8% | |
| Timed | 26 | 59.1% | |  | 60 | | | 96.8% | |
| Overflow | 26 | 59.1% | |  | 60 | | | 96.8% | |
| Gaits-Stations | 26 | 59.1% | |  | 60 | | | 96.8% | |

**Note:** SDT = Static detection threshold, DDT = Dynamic detection threshold, FFI = feedforward inhibition index, SMAD = simultaneous amplitude discrimination threshold, SSA = single-site adaptation, SMFD = simultaneous frequency discrimination threshold, SQFD = sequential frequency discrimination threshold, LII = lateral inhibition index.

**Supplementary Table 2. Mediation Sensitivity Analysis for Average Causal Mediation effect**

| Mediation model | Rho at which indirect effect (ab) = 0 |
| --- | --- |
| SM1 Glx -> SEQ Hyper-reactivity Score -> Total PANESS Score | 0.50 |
| SM1 Glx -> SEQ Hyper-reactivity Score -> PANESS Timed Score | 0.40 |
| SM1 Glx -> SEQ Hyper-reactivity Score -> PANESS Overflow Score | 0.40 |
| SM1 Glx -> SEQ Hyper-reactivity Score -> PANESS Gaits and Stations Score | 0.45 |
| SM1 GABA -> SEQ Hypo-reactivity Score -> Total PANESS Score | 0.50 |
| SM1 GABA -> SEQ Hypo-reactivity Score -> PANESS Timed Score | 0.50 |
| SM1 GABA -> SEQ Hypo-reactivity Score -> PANESS Overflow Score | 0.45 |
| SM1 Glx -> SEQ Hyper-reactivity Score -> PANESS Gaits and Stations Score | 0.40 |
| Thal GABA -> SEQ Seeking Score -> Total MABC-2 Score | -0.45 |
| Thal GABA -> SEQ Seeking Score -> MABC-2 Aiming and Catching Score | -0.40 |
| Thal GABA -> SEQ Seeking Score -> PANESS Overflow Score | 0.40 |

**References**

1. He JL, Oeltzschner G, Mikkelsen M, Deronda A, Harris AD, Crocetti D, et al. Region-specific elevations of glutamate + glutamine correlate with the sensory symptoms of autism spectrum disorders. Transl Psychiatry. 2021;11(1):411.

2. Harris AD, Puts NA, Edden RA. Tissue correction for GABA-edited MRS: Considerations of voxel composition, tissue segmentation, and tissue relaxations. J Magn Reson Imaging. 2015;42(5):1431-40.

3. Wolpert DM, Ghahramani Z, Jordan MI. An internal model for sensorimotor integration. Science. 1995;269(5232):1880-2.

4. Proske U, Gandevia SC. The proprioceptive senses: their roles in signaling body shape, body position and movement, and muscle force. Physiol Rev. 2012;92(4):1651-97.

5. Franklin DW, Wolpert DM. Computational mechanisms of sensorimotor control. Neuron. 2011;72(3):425-42.
